# Finite element and microstructural analyses indicate that pteraspid heterostracan oral plate microstructure was adapted to a mechanical function

**DOI:** 10.1111/pala.12733

**Published:** 2024-11-20

**Authors:** Madleen Grohganz, Antonio Ballell, Emily J. Rayfield, Humberto G. Ferron, Zerina Johanson, Philip C.J. Donoghue

**Affiliations:** 1 Bristol Palaeobiology Group, School of Earth Sciences, University of Bristol, Life Sciences Building, Tyndall Avenue, Bristol BS8 1TQ, UK; 3 Natural History Museum, Cromwell Road, London SW7 5BD, UK

**Keywords:** heterostracans, feeding, mechanical function, remodelling, finite element analysis (FEA), microstructure

## Abstract

Early vertebrate evolution has been characterized as a gradual shift from passive to more active feeding modes. However, this evolutionary scenario is contingent on poorly constrained inferences of the feeding ecology of extinct stem-gnathostomes. Heterostracans are among the earliest members of the gnathostome stem-lineage. Pteraspidiform heterostracans possessed an oral apparatus composed of rod-like plates that have been alternately interpreted to have been used for passive suspension feeding or mechanical food processing. Direct tests of the suspension feeding interpretation are challenging and so we tested hypotheses of a mechanical function using a combination of microstructural and finite element analysis (FEA). Our results demonstrate a negative relationship between simulated negative minimum principal stress (compressive stress) and bone volume fraction (a proxy for internal microstructure); the higher the stress, the higher the bone volume fraction. This relationship is clearest in the oral plate shaft. The hook, where the load is applied, shows the highest bone volume fraction values. Our results are compatible with adaption of skeletal microstructure to a mechanical function in which bone adaptively remodels under applied load to become denser to withstand increased stress. On this basis we reject the suspension feeding hypothesis in favour of a mechanical function for oral plates, such as deposit feeding or scavenging, in support for which we observe wear patterns on the aboral surface and the distal tip of the hook compatible with repeated abrasive polishing.

The New Head (Gans & Northcutt, 1983) and New Mouth (Mallatt, 1996) hypotheses posit that early vertebrate evolution is characterised by increasingly active food acquisition, from passive suspension feeding towards active predation over time. Living jawless vertebrates are a small and unrepresentative vestige of a much greater diversity and disparity of the fossil jawless ‘ostracoderms’ that dominated early vertebrate communities (Donoghue & Keating, 2014). The ostracoderms are phylogenetic intermediates of the living jawless and jawed vertebrates and they have been influential in informing on the gradual evolutionary assembly of the gnathostome body plan (Donoghue & Keating, 2014; Donoghue & Purnell, 2005; Janvier, 2001). Unfortunately, their feeding ecology is poorly constrained.

The extinct jawless heterostracans are among the earliest ostracoderms, both phylogenetically and stratigraphically (Blieck, 1984; Donoghue et al., 2000; Halstead, 1973; Keating et al., 2015). The feeding ecology of heterostracans has mainly been investigated in the pteraspids (Pteraspidiformes) which have several small, narrow, rod-like oral plates at the ventral margin of their mouth, organised in a symmetrical V-shaped arrangement (Stensiö, 1964; Dearden et al., 2024) (Fig. 1A and 1B). The individual oral plates vary in morphology, from approximately symmetrical median plates to more strongly asymmetrical plates at the margins of the apparatus. All the oral plates have a proximal shaft and a distal hook. In the lateral plates the shaft is proportionally longer than the hook (Fig. 2A and 2B), whereas in the short median plates the hook and the shaft are about equal in length (Fig. 3A and 3B). With their shaft the oral plates (except for the median plates) articulate directly with the postoral plate (Fig. 1A).

Hypotheses on the feeding mode of heterostracans range from suspension feeding to mechanicaI feeding. Several authors argue for a suspension feeding interpretation (Janvier, 2015; Lingham-Soliar, 2014; Stiefel, 2021; Dearden et al., 2024) in which the heterostracans opened and closed their orifice using the denticulated oral plates to filter particles from the water column. Other arguments for a passive feeding mode and against an active predatory lifestyle have been based on proposed functional constraints due to the lack of jaws in heterostracans (Halstead, 1973; Romer, 1959, 1966, 1970). However, this view is effectively refuted by the occurrence of macrophagous feeding in jawless lampreys, hagfish and conodonts (Gans & Northcutt, 1983; Purnell, 1995).

Other authors have proposed a mechanical function for the oral plates. Kiaer (1928) argues for predation, with the oral plates actively biting or crushing prey, analogous to gnathostome jaws. A scavenging feeding mode has been hypothesized, with the oral plates reconstructed as having been arranged in a manner similar to the hagfish feeding apparatus (Janvier, 1974; Jarvik, 1980; Stensiö, 1932). Herbivorous macrophagy has also been proposed with the oral plates of *Anglaspis* (Cyathaspidiformes) envisaged to have been used as teeth, shearing against each other to snip algal fragments (Bendix-Almgreen, 1986). The mouth of *Pteraspis* has also been interpreted as protrusible, used for selective detritus feeding with the oral plates acting as a sediment scoop (White, 1935). Evidently, interpretations of heterostracan feeding ecology are in a state of flux and the majority of them have yet to be tested, relying instead on poorly constrained inferences based on morphological analogy with extant jawed or jawless vertebrates.

Here we test for a mechanically-intensive function of the oral plates using finite element analysis (FEA) and microstructural analysis. Active feeding modes should lead to higher and non-uniform distribution of stress within the oral plates compared to passive feeding behaviours. Therefore, our hypothesis predicts changes in the internal microstructure of the oral plate in areas where high stresses concentrate in response to active feeding loads.

## Materials and Methods

In our experiments, we test a mechanically-intensive function for the tooth plates of pteraspid heterostracans. We based our analyses on isolated plates of *Loricopteraspis dairydinglensis*, collected from the Lower Devonian, Ditton Group, Dairy Dingle locality near Neenton, Shropshire, UK (Ball & Dineley, 1961) (specimen NHMUK PV P 43711) and *Pteraspis* sp., collected from the Lower Devonian, Ditton Group, New Inn 2 locality, near Upper Hayton, Shropshire, UK (Ball & Dineley, 1961) (specimen NHMUK PV P 76697). We focus on these taxa because isolated oral plates from heterostracans are generally rare and oral plates from these taxa have been the focus of previous analyses of oral plate function in heterostracans (Grohganz et al., 2023; Purnell, 2002). All examined material is housed in the Natural History Museum, London (NHMUK).

We adopt the following terminology (Dearden et al., 2024) to describe the nature and arrangement of the oral plates: we use lateral/median to describe the position of the oral plates in the apparatus, from those located towards the lateral margins to those located towards the middle of the apparatus; oral/aboral to describe the direction of plate surfaces relative to the oral cavity, the oral surface with open vascular canals facing the inside of the oral cavity and the aboral surface with tubercles facing the outside of the oral cavity; proximal/distal to refer to the position on the oral plates relative to their junction with the postoral plate, proximal as located close to this junction and distal as located far from it. Previously proposed mechanical functional hypotheses describe the hooks as working against the rostrum in a biting scenario (Kiaer, 1928) or being used for scooping sediment (White, 1935). In these mechanical scenarios, a load is applied to the hook of the oral plate either through contact with hard sediment or food. We test whether the internal microstructure of different morphologies (lateral and median) in the heterostracan oral plate apparatus are adapted to a mechanical function. For this we use two sets of data based on computed tomographic scans of heterostracan oral plates, a) the stress value predictions from FEA simulations and b) bone volume fraction value calculations.

If the heterostracan oral plates are adapted to a mechanical function, we should expect to see a correlation between simulated stress and microstructure. Following Wolff’s Law, high stress develops in areas of the bone where high functional forces are applied (Wolff, 1892). As a response to the imposed load, the bone strengthens and adaptively remodels in the form of increased bone volume as well as increased bone density (Currey, 2002; Dalén & Olsson, 1974; Goodship et al., 1979; Johnson et al., 2000; Jones et al., 1977; King et al., 1969; Lanyon et al., 1982). Remodelling is generally defined by adding or removing bone; to quantify these processes in our specimens we focus on bone density (bone volume fraction) as a proxy. Based on the principle of adaptive bone remodelling, we anticipate higher bone volume fraction values/higher bone density in areas with higher simulated stress. If the oral plates simply closed the mouth or were used in suspension feeding and lacked a mechanically-intensive function, we would not anticipate structural adaption to significant loads.

### Model creation

We prepared two 3D models representing the two principal plate morphologies present in the pteraspid heterostracan oral apparatus: one model of a lateral oral plate located towards the side of the apparatus (Fig. 2; specimen NHMUK PV P 43711) and another of a median oral plate located towards the middle of the apparatus (Fig. 3; specimen NHMUK PV P 76697). We examined these two oral plate morphologies separately in our analyses as we hypothesize that they might perform different functions within the apparatus during feeding. 3D models are based on tomographic models of oral plates from *Loricopteraspis dairydinglensis* and *Pteraspis* sp.. The tomographic data were obtained using Synchrotron Radiation X-ray Tomographic Microscopy (srXTM; Donoghue *et al*. 2006) (see Data S1 for further details). Virtual models were created in Avizo Lite v 9.5.0 (https://www.fei.com/software/avizo3d) and exported as stereolithograph files (Fig. 2 and Fig. 3). The models were imported into Blender v2.92.0 (https://www.blender.org) and subjected to scaling to test the effects of shape without invoking size effects, appropriate when comparing bone volume fraction to the distribution of stress. We used a Python script (see deposited data) to rescale the models to the same (arbitrary) surface area of 100 cm^2^ for model comparison by equal force to surface area-scaling. The surface area-scaling approach removes the effect of model size and has been used as the standard procedure in palaeobiology applications of FEA (Dumont et al., 2009). Later, a volume-scaling approach has been introduced (Fortuny et al., 2015; Marcé-Nogué et al., 2013). We additionally carried out volume-scaling by adjusting the applied force for the median oral plate model (using Equation 6 from Fortuny et al., 2015). In the results section we compare the outcomes of the surface area-scaling to the volume-scaling approach.

We used the mesh modifier’s voxel remesher tool in Blender to remesh the surface mesh. The triangulate modifier tool was applied to generate triangular surface elements. The surface of the oral plates was smoothed in sculpt mode in Blender to create an idealised and more generalised model focusing on the overall shape of the oral plate rather than its surface ornamentation. Surface ornamentation details differ between various groups of heterostracans but the general oral plate morphology that this model represents is relevant to a broader range of heterostracan taxa. As such, the results are generalisable across the entire group of pteraspids which share the same overall oral plate morphology. Mesh checks were performed to make sure the mesh had a closed and clean surface (manifold) and that each edge has exactly two incident faces. The Blender models were then exported in meters unit as stereolithograph files for further processing in Hypermesh 2022 (Altair, https://www.altair.de/hypermesh/) where we used the tetrameshing function to create a 3D mesh of tetrahedral elements and to check the resulting mesh quality. We use a solid model, that is completely filled with tetrahedral mesh elements, and we do not incorporate the internal structure of the oral plates into these models. Our intention is to use the FE-simulation to predict where bone remodelling may occur (high bone volume fraction would be predicted in areas of high stress and low bone volume fraction in areas low stress). This offers a prediction for bone remodelling in the internal structure of the heterostracan oral plates related to stress patterns. In a FE model that incorporates microstructure, stresses are expected to be homogeneously distributed throughout the structure. No gradients in stress should be observed, as the internal structure is assumed to be adapted to the mechanical function and stresses already normalized. Therefore, keeping the FEA analysis separate from the internal structure and using a solid model has the highest predictive power.

The meshes were then imported as .inp models into Abaqus v. 6.14-1 (Simulia, https://www.3ds.com/products/simulia/abaqus), where they were assigned the elastic, isotropic, and homogeneous material properties of acellular bone (in fish) with a Young’s modulus of 6480 x 10^6 Pa (based on measurements on the rib bones of great sculpin) (Horton & Summers, 2009) and a Poisson’s ratio of 0.3 (based on measurements on the rib bones, vertebrae, mandible and operculum of brown trout, flounder and sea bass) (Grenoble et al., 1972). Assigning exact material properties for the heterostracan oral plates is difficult as there are no appropriate extant analogues for oral plates and fossilisation processes transform original material properties. For our analyses we assumed acellular bone because heterostracan aspidin has been shown to be a type of acellular dermal bone (Moss, 1961; Ørvig, 1965; Stensiö, 1927), the spaces within trabeculae having housed unmineralized collagen fibre bundles (Keating et al., 2018). Acellular bone is capable of mechanosensing and can remodel (Currey et al., 2017; Witten & Huysseune, 2009).

Comparative analysis of the histology of the internal structure of the heterostracan dermal skeleton and the oral plates has shown profound similarities in the general tissue arrangement and the included layers (Keating et al., 2015). Thus, we used for our analyses the material properties of acellular bone for the heterostracan oral plates.

### Finite element analysis

Three-dimensional FE analyses were performed in Abaqus. We performed convergence tests using the lateral oral plate model as a reference to ensure that results are independent of changing mesh element numbers chosen in Blender (see deposited data). A solution was considered independent if the converged value for the von Mises stress did not change by more than 5% between a simulation and the next. We compared results for the arithmetic mean and the mesh-weighted arithmetic mean (MWAM, accounting for element size differences within non-uniform meshes) (Marcé-Nogué et al., 2016) using R v4.2.2 (R CORE TEAM, 2013) (see deposited data for the R code, based on that of Ballell and Ferron, 2021). Convergence is generally faster for the MWAM than for the AM. The analyses show that results are independent of the number of mesh elements chosen for every tested mesh with element numbers of 25,000 or higher for the MWAM and 50,000 or higher for the AM (see Data S2). Therefore, we chose a 50,000 element mesh for both the lateral and median plate analyses, as it tends to show the best trade off in terms of consistency of results and computational time.

Boundary conditions were applied by constraining all nodes at the proximal end of the oral plate in all degrees of freedom; we assumed our models to be static in translation and rotation. For this we created a multi-point constraint consisting of a set of constraint nodes on the proximal end of the oral plate, which we linked to a reference node (floating in space close to the mesh, posterior RP), to which the boundary condition was applied (Fig. 4A, upper image).

FEA was performed with a load applied to a set of nodes defining the ridge of the oral plate hook to simulate mechanical use of the oral plate (Fig. 4A, upper image). In the surface area-scaled models (both the lateral and median oral plate), a magnitude of 10 N was applied in - y direction to the reference node (floating in space close to the mesh, biting RP), which uniformly transferred the load to the linked nodes on the oral plate hook. For the volume-scaling approach, we used the same models, but applied a volume corrected force with a magnitude of 10.2 N in -y direction to the median oral plate. For an estimation of potential heterostracan bite force we followed estimations of bite force based on recent jawless fish (i.e. hagfish) (Clark & Summers, 2007). However, estimating heterostracan bite force is difficult, as no recent analogues of the oral plates are available for comparison. Therefore, we also ran analyses with a bite force of 100 N (see deposited data). As expected, the magnitude of the stress outputs changes, but not the overall pattern of stress direction and distribution (see Data S3). As our analyses mainly focus on differences in stress distribution and not actual numerical stress values, our results mean that we can assume a bite force magnitude of 10 N for our analyses.

In addition, simulations with several angles of attack (0 deg, 15 deg, 30 deg and 45 deg) were performed to check for potential differences in the overall stress pattern depending on the angle (see Data S3). Our results show no substantial changes in the stress distribution pattern with different angles of attack except for a relocation of areas of moderate minimum principal stress from the proximal to the distal end of the hook. So, we used a model of 0 deg angle of attack for all analyses. FEA results were summarised in field outputs including minimum principal stress, maximum principal stress, and Von Mises stress, a commonly used parameter in palaeobiology (Rayfield, 2007), predicting failure under ductile fracture (Dumont et al., 2009). Abaqus outputs are unitless and therefore the output unit of the stress results depends on the input units of the other variables. Here, all stress values were output in Pa following from the input units of the other variables (load in N, length in m, Young’s modulus in Pa=N/m^2; in case the length is input in mm, Young’s modulus input is in MPa=N/mm^2 and the stress output is also in MPa). Minimum principal stress and maximum principal stress were visualised as colour plots and vectors showing the spatial distribution as well as direction of these parameters. For minimum principal stress, we used only the negative values, that represent the compressive component of our stress results and for maximum principal stress we used only the positive values, that represent the tensile component. Increasingly negative values indicate increasing compressive stress, whereas increasingly positive values indicate increasing tensile stress. For visualisation we used the inferno colour map, which has been shown to increase accessibility and reduce data distortion compared to the standard rainbow colour map (Lautenschlager, 2021).

### Correlation of stress parameters and internal growth structure

To investigate a potential correlation between stress parameters and the internal growth structure of the oral plates, we compared the results of the FEA analyses with bone volume fraction values derived from tomographic scans. Analyses were conducted on matched 3D volumes of longitudinal (z plane) cuts through the FE model and the tomographic models. Compression is mainly withstood by bone, which is stronger under compressive stress than under tension (Bayraktar et al., 2004; Beaupied et al., 2007; Keaveny et al., 1994). Soft tissues like tendons or cartilage will rather withstand tensile stress and strain (Holzapfel, 2001; Laasanen et al., 2003; Summers & Koob, 2002). In pteraspid oral plates the proposed soft tissue cover of the oral surface (see Dearden et al., 2024; and our own observations in the results) would have mainly withstood the tensile stress component, while the bone of the oral plates would have mainly withstood the compressive stress component. For our analyses and the stress-bone microstructure comparison, we therefore focus on the compressive stress component only, which is represented by the negative values of our minimum principal stress results.

### Extracting stress parameters from the Abaqus FEA analyses

We created a longitudinal z plane “on cut” slice (selected under view cut manager in Abaqus) through the middle of the FE model (Fig. 4A, lower image). The location of the middle/cut line was determined as the mean value of the minimum and maximum nodal z values of the whole coordinate set of the sample in Abaqus. The thickness of the “on cut” slice was set to 3.4% of the whole sample z length left and 3.4% of the whole sample z length right of the middle line. This value was chosen, as it accounts for the volume with the highest density of nodes around the middle cut. These percentage values were also adapted for the median plate to make the analyses more comparable. From this z plane “on cut” in Abaqus we exported for every node the stress values (minimum principal stress) and respective coordinates (Fig. 4B).

### Extracting bone volume fractions from the tomographic data

Virtual z plane slices through the oral plate tomographic model were created in VGStudioMax v3.5.1 (https://www.volumegraphics.com) (see deposited data). A downsampled image stack of 60 images was imported into Fiji (Schindelin et al., 2012) (https://fiji.sc). The z plane middle cut corresponding to the middle cut through our FEA Abaqus model was determined. From this middle cut through the tomographic image stack, we took two images in the z direction and two images in the -z direction. The number of images corresponds to 3.4% of the total amount of images in the stack left and 3.4% of total amount of images in the stack right of the middle cut. This is also the value we used to subsample the Abaqus coordinates, which ensures we are correlating the same volumes from our FEA analyses (stress values) and the tomographic images (bone volume fraction).

The resulting image stack of 5 tomographic slices was converted into binary images using the threshold command in Fiji (Fig. 4C) (see deposited data). The slices were converted into a raster stack using the raster package v3.6-3 (Hijmans, 2022) stack function in R. Every layer in the stack was subsequently split into 25x25 rectangular sub images using a custom-built R function (see deposited data) (Fig 4D).

We matched each of the 25x25 images per layer in the stack of raster images to the respective coordinates and their associated stress values extracted from the FEA analyses (Fig. 4B and D) (see deposited data). Based on the subdivided raster image stack, bone volume fraction values were calculated for every raster volume throughout the whole stack of 5 images using the volume fraction function of the BoneJ2 plugin (Domander et al., 2021) in Fiji (see deposited data) (Fig. 4E). A subset of negative minimum principal stress values was created to focus the analyses on the compressive stress component. Bone volume values and respective minimum principal stress values were then calculated as mean values for every raster. We omitted the proximal-most region of the oral plate for visualisation and analysis, as the model was constrained at this region. Constraints can lead to erroneously high localised stress values, which were not used for further analysis. For minimum principal stress we excluded raster volumes with values < -300,000 Pa (for the lateral oral plate) (see Fig. 6A) and < -100,000 Pa (for the median oral plate) (see Fig. 6B). For bone volume fraction we omitted raster volumes with values <0.2 from further analyses (see Fig. 6E and F), as they mostly include rasters at the margin of the sample and include empty space, which distorts the bone volume analysis. This helps to constrain our analysis to the interior of the oral plates, where we hypothesise remodelling to occur. Subsetting for these parameters leads to different shapes of the rastered results for minimum principal stress and bone volume fraction values (compare e.g. Fig. 6A and E). Thus, correlation tests were performed only for raster volumes, for which both minimum principal stress values and bone volume fraction values were available. We used the stats package cor.test function in R to test for a correlation between minimum principal stress raster means and bone volume raster means. To investigate spatial autocorrelation between adjacent raster volumes and calculate an adjusted correlation coefficient we additionally performed Dutilleul's modified t-test (Dutilleul et al., 1993) using the SpatialPack package modified.ttest function (Vallejos et al., 2020). All statistical analyses and visualisations were performed in R (see deposited data).

## Results

### General oral plate morphology and wear patterns

**Fig. 1:**
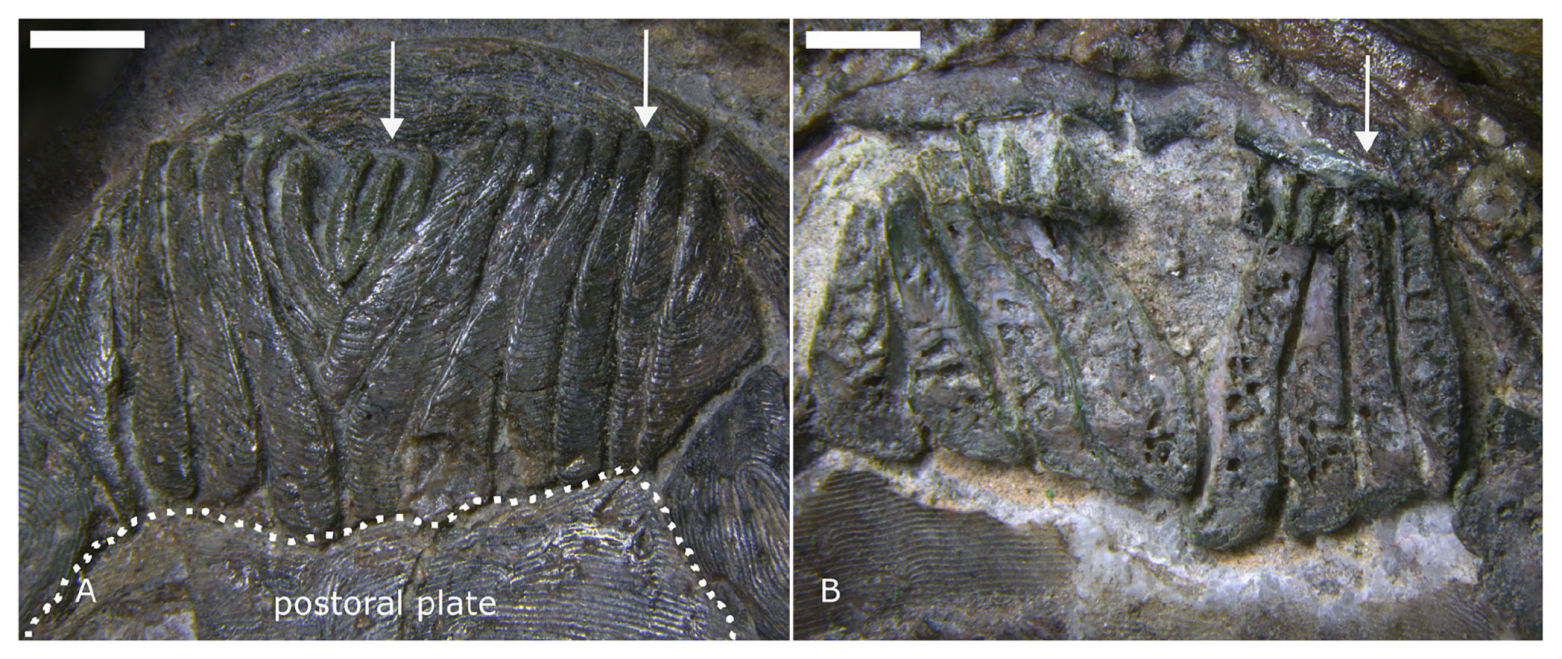
Articulated, V-shaped oral plate apparatus of *Protopteraspis vogti* (Kiaer, 1928) (specimen PMO A28720; Lower Devonian, Red Bay series, NW slope (300-400 m) at Ben Nevis, Raudefjorden, Spitsbergen, NO): A, aboral view; B, oral view. Arrows mark individual median (left) and lateral (right) oral plates. Scale bars represent 2 mm.

**Fig. 2:**
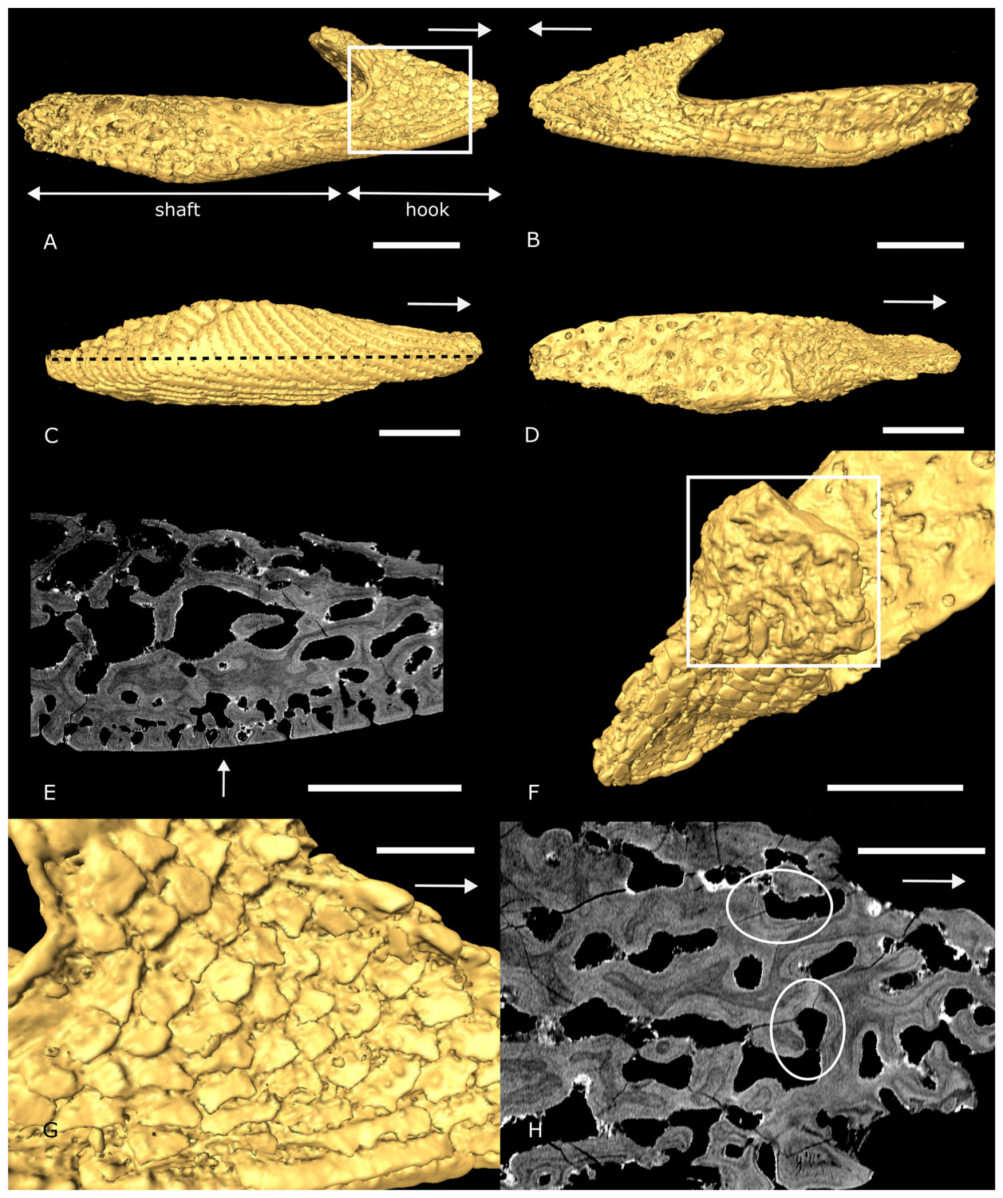
3D surface models and tomographic sections of *Loricopteraspis dairydinglensis* lateral oral plate (specimen NHMUK PV P 43711; Lower Devonian, Ditton Group, Dairy Dingle locality (Ball & Dineley, 1961), near Neenton, Shropshire, UK): A-B, lateral view, rectangle in A indicates position of close-up in G; C, aboral view, line represents position of tomographic section in E; D, oral view; E, tomographic section through the oral plate shaft showing abraded tubercles on the aboral surface (arrow); F, close-up of oral hook surface with abrasion on the distal tip (rectangle); G, close-up of denticles on the lateral hook surface shown in A; H, tomographic section through the hook showing remodelling of the internal microstructure (circles). Arrows indicate distal direction, scale bars represent: 1000 microns (A-D, F); 500 microns (E); 300 microns (G); 200 microns (H).

The lateral oral plate is located towards the lateral margin of the oral plate apparatus (Fig. 1A and 1B). Its proximal part, which articulated against the dermal skeleton, is comprised of a long shaft, which forms most of the proximal-distal oral plate length (Fig. 2A, see also Fig. 4A, posterior RP). The distal part has a hook-like process facing the oral cavity; this is where the load is applied in our analyses (see Fig. 2A).

The lateral oral plate model shows signs of wear in the form of abrasion of the tubercles on its outer (aboral) surface, especially around the middle of the shaft (Fig. 2C and 2E, arrow). We also observe abrasion of the denticles on the lateral faces of the oral plate (Fig. 2G) as well as on the distal tip of the oral plate hook (Fig. 2F, rectangle). In the hook region we find evidence of remodelling of the internal microstructure with vascular spaces truncating existing growth layers (Fig. 2H, circles).

**Fig. 3:**
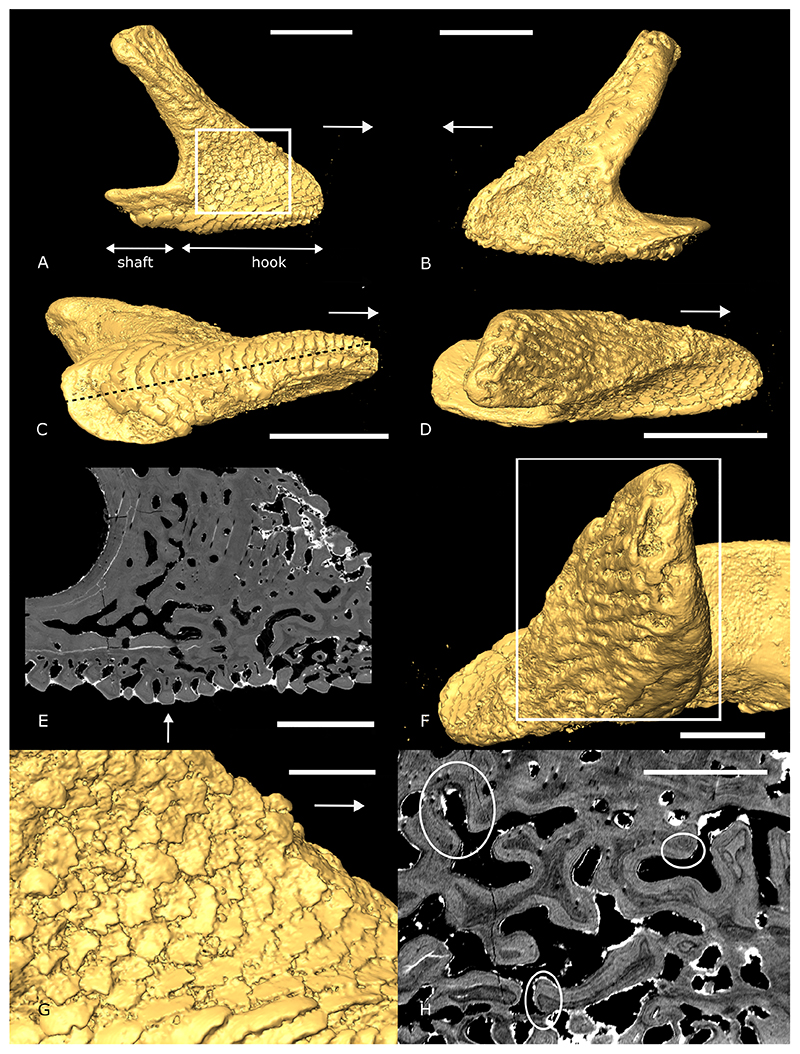
A-H, 3D surface models and tomographic sections of *Pteraspis* **sp**. median oral plate (specimen NHMUK PV P 76697; Lower Devonian, Ditton Group, New Inn 2 locality (Ball & Dineley, 1961), near Upper Hayton, Shropshire, UK): A-B, lateral view, rectangle in A indicates position of close-up in G; C, aboral view, line represents position of tomographic section in E; D, oral view; E, tomographic section through the oral plate shaft showing non-abraded tubercles on the aboral surface (arrow); F, close-up of oral hook surface with abrasion on distal tip (rectangle); G, close-up of denticles on the lateral hook surface shown in A; H, tomographic section through the hook showing remodelling of the internal microstructure (circles). Arrows indicate distal direction, scale bars represent: 1000 microns (A-D); 500 microns (E-F); 300 microns (G); 200 microns (H).

**Fig. 4:**
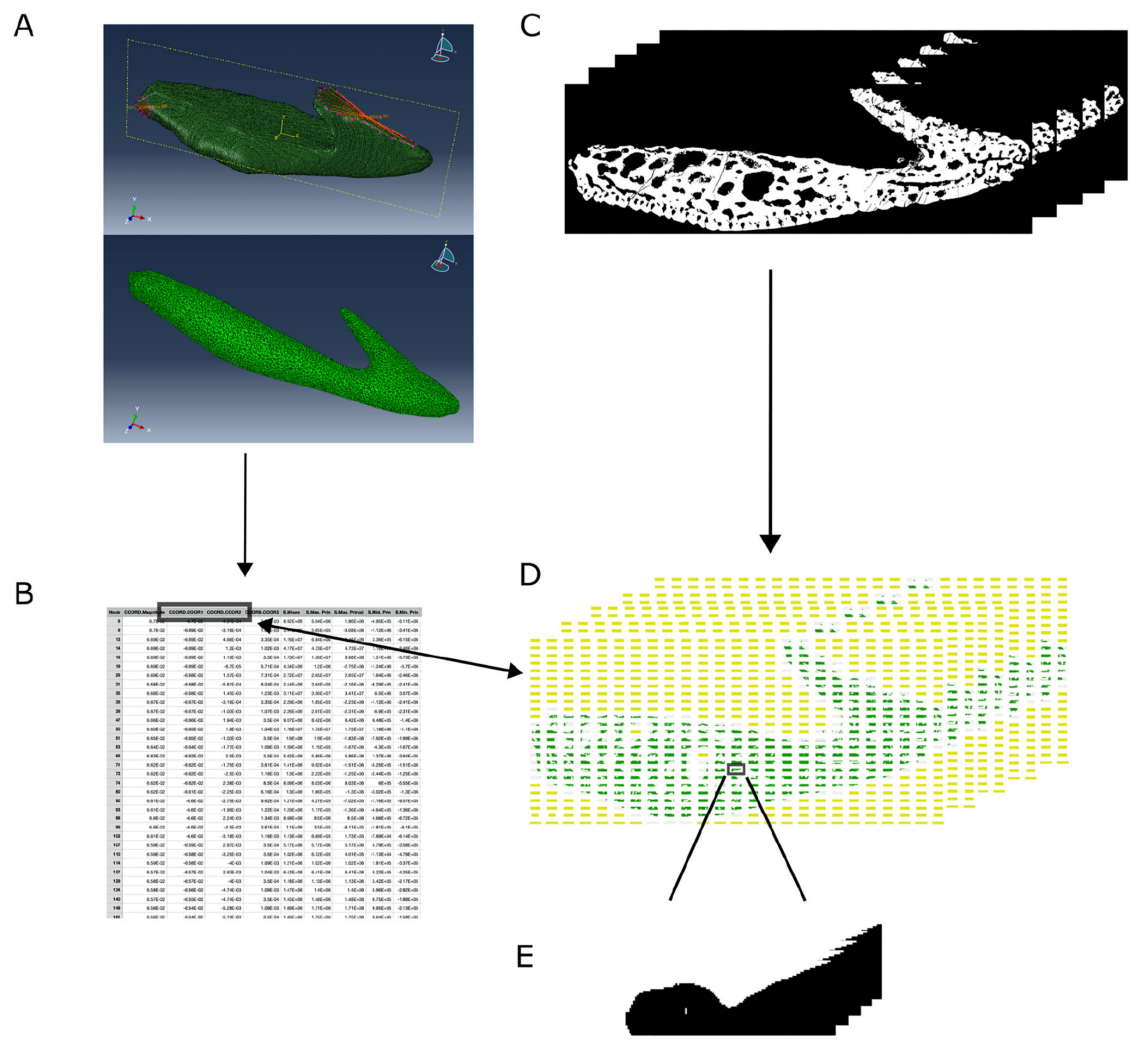
Workflow for correlating stress parameters and internal growth structure: A, upper: 3D FE model of the lateral oral plate showing location of set of slave nodes at the proximal end of the oral plate linked to master node ‘posterior RP’ and set of slave nodes at the oral plate hook linked to master node ‘biting RP’, to which the load was applied in our FEA simulations, lower: creating a longitudinal z plane “on cut” slice in Abaqus; B, exporting stress values (minimum principal stress) and respective coordinates for every node in the z plane “on cut” slice; C, creating virtual z plane slices through the oral plate tomographic model and converting the slices into binary images; D, converting the slices into a stack of raster layers and splitting every raster layer into 25x25 rectangular sub images; E, calculating bone volume fraction values for every raster volume throughout the whole image stack.

The median oral plate is located towards the middle of the oral plate apparatus (Fig. 1A). Its proximal part (shaft) is much shorter in comparison to the lateral plate and the distal part comprises a proportionally larger hook (Fig. 3A). Compared to the lateral oral plate the median oral plate model does not show wear on the aboral side; the tubercle structures are not abraded (Fig. 3C and 3E). The denticles on the lateral surfaces of the median oral plate are also less abraded than the denticles on the lateral plate (Fig. 3G). The former denticles resemble more closely the sharp pointed denticles described by Purnell (2002: fig. 2c, d). The distal tip of the oral plate hook shows signs of abrasion (Fig. 3F, rectangle). In the median oral plate hook we also observe evidence of remodelling in the internal microstructure in the form of vascular spaces truncating existing growth layers (Fig. 3H, circles).

### Stress patterns

**Fig. 5:**
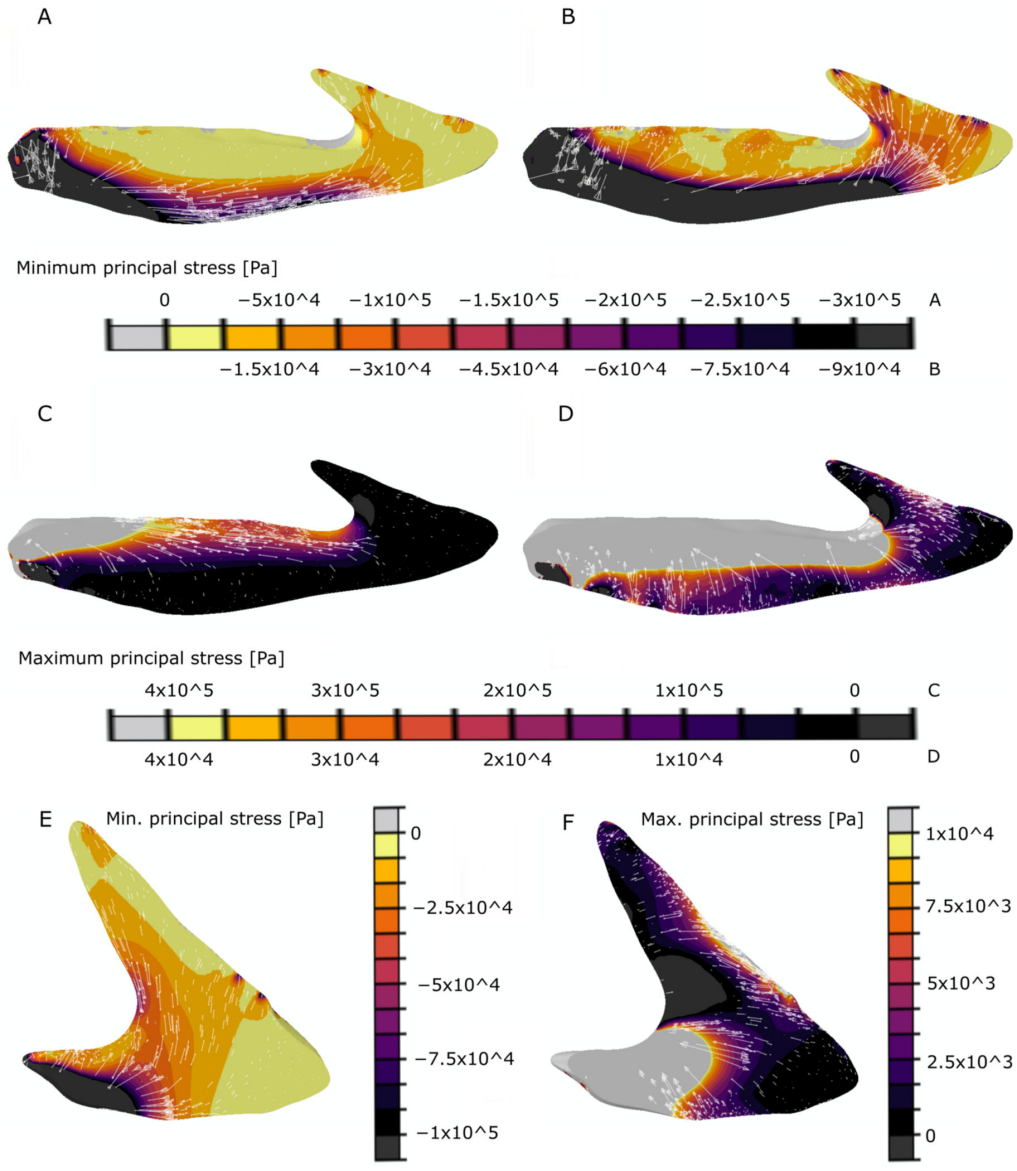
A-D, results of the FE analyses of lateral oral plate sample NHMUK PV P 43711: A-B, minimum principal stress patterns, with minimum principal stress increasing towards the right of the legend; C-D, maximum principal stress patterns, with maximum principal stress increasing towards the left of the legend; E-F, results of the FE analyses of median oral plate sample NHMUK PV P 76697: E, minimum principal stress patterns, with minimum principal stress increasing towards the bottom of the legend; F, maximum principal stress patterns, with maximum principal stress increasing towards the top of the legend. With stress trajectories as arrows. Legends below/beside respective graphs. The visualisation threshold for maximum and minimum principal stress values was set manually to better image the remaining stress patterns as well as to exclude exceedingly high stress values at the proximal end of the oral plate, which can be attributed to the fact that the model is fixed in this area.

Here we describe the results of the FEA analyses of the surface area-scaled oral plate models (Fig. 5). We compare them to the results of the volume-scaled model, which can be found in the supporting information (Data S4).

In the surface area-scaled model of the lateral oral plate the highest minimum principal stress occurs in the shaft on the aboral side (Fig. 5A). There, minimum principal stress trajectories are generally oriented parallel to the aboral margin and absolute stress values decrease from the aboral to the oral margin. Maximum principal stress values show the opposite pattern, with highest absolute values in the oral part of the shaft decreasing aborally (Fig. 5C). The main stress components in the proximal shaft are compression in the aboral region (represented by negative minimum principal stress values) and tension in the oral region (represented by positive maximum principal stress values) leading to bending of the sample when a load is applied in the distal hook-like structure.

In the distal hook, minimum principal stress occurs approximately in parallel (Fig. 5B), maximum principal stress approximately perpendicular (Fig. 5D) to the direction of the load applied. Minimum principal stress as well as maximum principal stress values stay relatively constant throughout the distal hook on a medium to low level.

In the surface area-scaled model of the median oral plate, the stress patterns in the distal hook are comparable to those observed in the lateral plate, minimum principal stress occurs approximately in parallel (Fig. 5E), maximum principal stress approximately perpendicular (Fig. 5F) to the direction of the load applied on the distal hook. Minimum principal stress as well as maximum principal stress values remain approximately constant throughout the distal hook on a medium to low level (Fig. 5E and 5F), with spatial patterns comparable to the lateral oral plate hook (see Fig. 5A to 5D). For the analyses, the median oral plate model is fixed at the shaft, which generates exceedingly high stress values there. Hence, we excluded most of the stress values in the shaft as it is comparably short in the median oral plate.

In the volume-scaled model of the median oral plate the absolute stress values change only slightly in comparison the surface area-scaled model due to adjusting the value of the applied load (to 10.2 N). However, the general patterns of minimum principal stress (Data S4A) and maximum principal stress (Data S4B) remain the same as described above for the surface area-scaled median oral plate model (see Fig. 5E and F).

### Rastered stress and bone volume fraction values

**Fig. 6:**
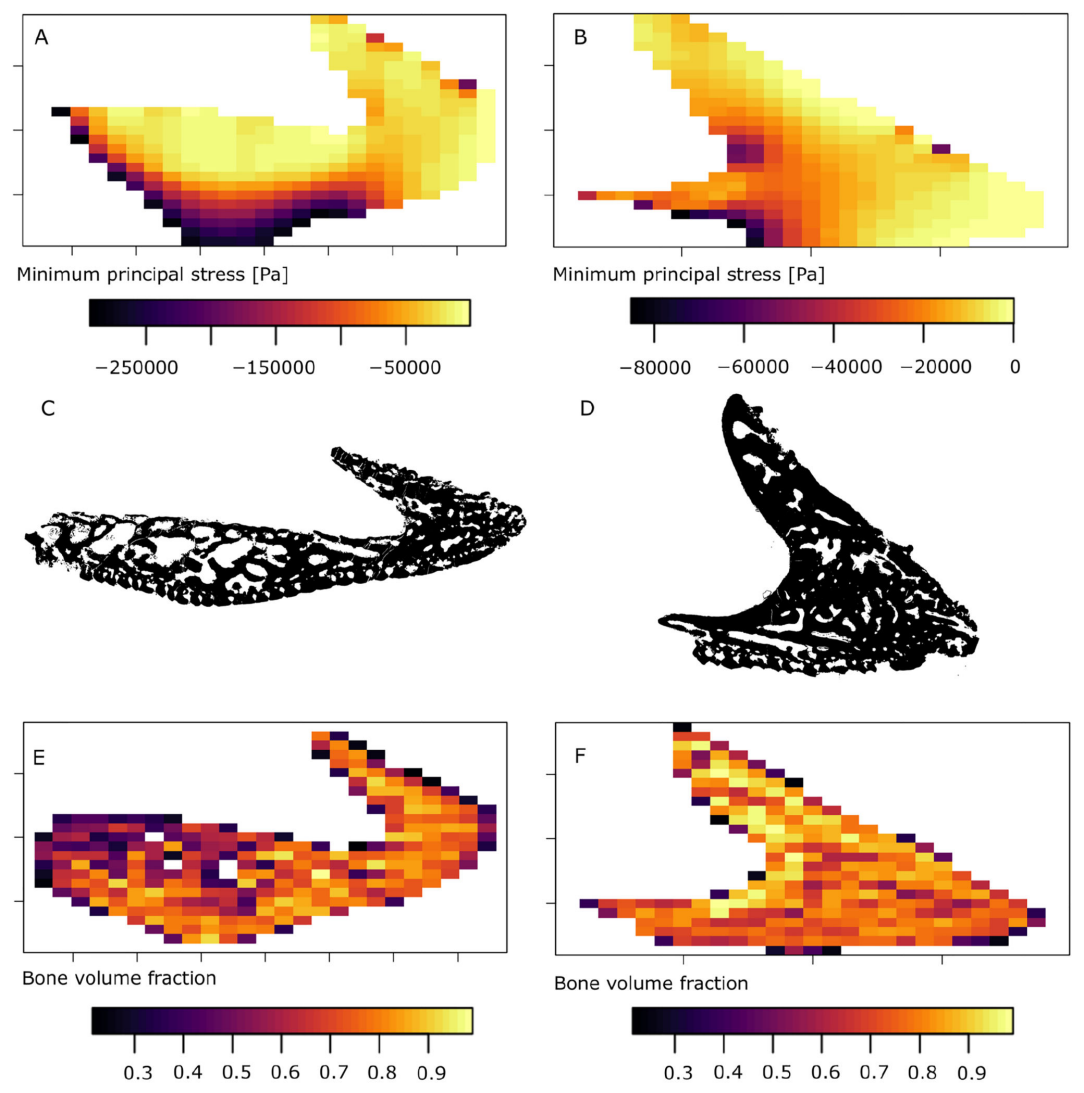
A, lateral oral plate sample NHMUK PV P 43711 rastered FEA results for minimum principal stress; B, median oral plate sample NHMUK PV P 76697 rastered FEA results for minimum principal stress; C, lateral oral plate sample NHMUK PV P 43711 binary image of tomographic z-plane cut; D, median oral plate sample NHMUK PV P 76697 binary image of tomographic z-plane cut; E, lateral oral plate sample NHMUK PV P 43711 rastered results for bone volume fraction values; F, median oral plate sample NHMUK PV P 76697 rastered results for bone volume fraction values.

In the surface area-scaled model of the lateral oral plate, the rastered predictions for negative minimum principal stress (compression) (Fig. 6A) match the original FEA results (Fig. 5A). They show a gradient from higher absolute stress values in the aboral part of the oral plate shaft to lower values in the oral part of the shaft and medium to low values in the hook.

The results for rastered bone volume fraction values (Fig. 6E) based on tomographic slices (Fig. 6C) show that in the hook region, bone volume fraction values remain approximately constant at a high level, including the highest overall bone volume fractions. In the proximal shaft of the oral plate, bone volume fraction shows a gradient comparable to the minimum principal stress predictions. Highest values occur in the aboral part of the shaft, decreasing orally (Fig. 6E).

In the surface area-scaled model of the median oral plate, the rastered predictions for negative minimum principal stress (compression) (Fig. 6B) match the original FEA results (Fig. 5E). This is also the case in the volume-scaled median oral plate model (Data S4C). The rastered bone volume fraction values (Fig. 6F) based on tomographic slices (Fig. 6D) show that throughout the median oral plate sample bone volume fraction values remain approximately constant at a high level. In contrast to the lateral oral plate, the shaft in the comparably short median oral plate does not show a gradient in bone volume fraction values.

### Relationship between bone volume fraction and minimum principal stress

**Fig. 7:**
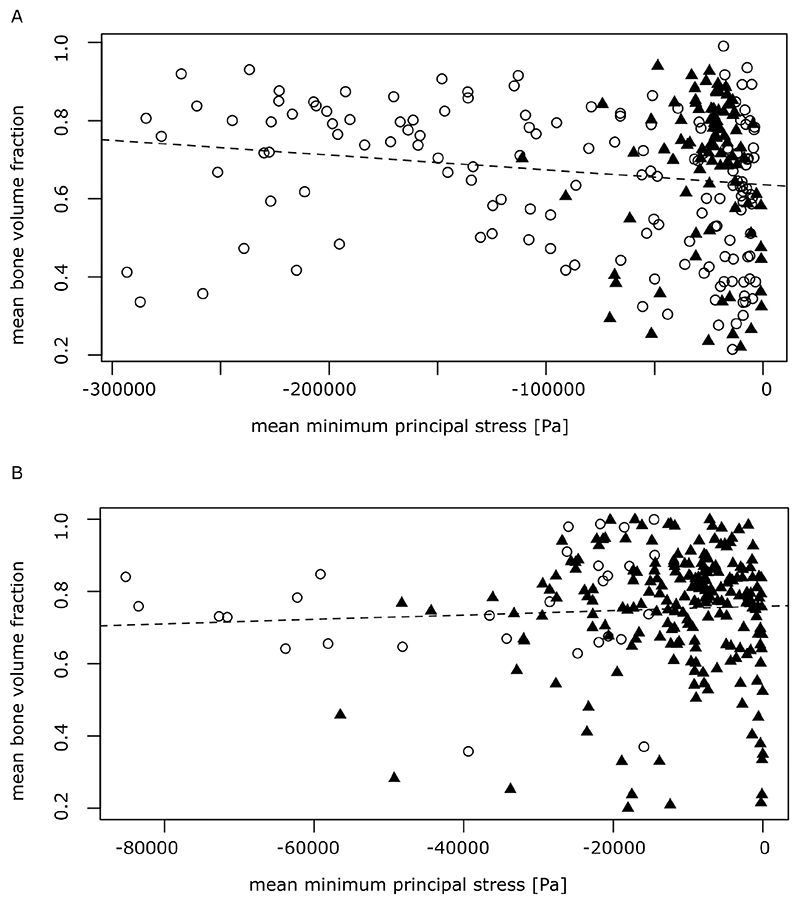
Bone volume fraction raster means plotted against minimum principal stress raster means: A, results for lateral oral plate sample NHMUK PV P 43711; B, results for median oral plate sample NHMUK PV P 76697. Circles indicate raster volumes in the shaft of the oral plate, triangles in the hook of the oral plate; the dotted line represents the linear relationship between the two variables, bone volume fraction and minimum principal stress.

We plotted the bone volume fraction raster means against negative minimum principal stress (compression) raster means for the surface area-scaled lateral (Fig. 7A) and median (Fig. 7B) oral plates. For the distal hook region in the lateral oral plate (Fig. 7A, triangles), the majority of points appear clustered. They show relatively low minimum principal stress values and high bone volume fraction values. For the proximal shaft region (Fig. 7A, circles), bone volume fraction values are more variable. But generally, the highest stress values are associated with the highest bone volume fraction values. Correlation analysis (non-parametric test, Spearman’s rho) confirms a significant negative correlation between negative minimum principal stress (compression) and bone volume fraction (p-value = 0.001267, rho ˜ -0.21) (deposited data). The higher the mean absolute minimum principal stress values, the higher the associated mean bone volume fraction value. Dutilleul’s modified t-test, correcting for autocorrelation, indicates a similar negative but non-significant correlation between negative minimum principal stress (compression) and bone volume fraction (p-value = 0.1587, cor ˜ -0.15) (deposited data).

For the distal hook in the median oral plate (Fig. 7B, triangles), most points appear clustered, as in the lateral plate. They show relatively low minimum principal stress values and mostly high bone volume fraction values. For the proximal shaft region (Fig. 7B, circles), only a few points could be plotted as a result of thresholding. For the surface area-scaled median oral plate model, correlation analysis (non-parametric test, Spearman’s rho) does not indicate a significant relationship between negative minimum principal stress (compression) and bone volume fraction (p-value = 0.7931 rho ˜ -0.02) (deposited data). Dutilleul’s modified t-test, which accounts for autocorrelation, also does not return a significant relationship (p-value = 0.6464, cor ˜ 0.05) (deposited data).

When plotting bone volume fraction raster means against negative minimum principal stress (compression) for the volume-scaled median oral plate model the general pattern is the same as in the surface area-scaled median oral plate described above (Data S4E).

Correlation analysis (non-parametric test, Spearman’s rho) returns very similar values (p-value = 0.7894, rho ˜ -0.02) to the surface area-scaled model and does also not indicate a significant relationship between negative minimum principal stress (compression) and bone volume fraction (deposited data). The same is true when accounting for autocorrelation with Dutilleul’s modified t-test (p-value = 0.6461, cor ˜ 0.05) (deposited data).

## Discussion

In the proximal shaft region of the lateral oral plate model, our analyses show that bone is densest towards the aboral surface where the minimum principal stress is highest. This correlation points towards an adaptation of the internal microstructure of the heterostracan oral plates to stresses resulting from mechanical loading; denser bone occurs in areas of higher simulated minimum principal stress. Maximum principal stress is highest towards the oral surface of the shaft (Fig. 5C). This surface was probably covered in soft tissue in life, which is supported by the observation of open vascular canals on the oral surfaces of the plates (Fig. 2D). The soft tissue cover of the oral surface of the plate probably withstood the high positive maximum principal stresses (tension) in this area, while the bone of the oral plate withstood the high negative minimum principal stresses (compression).

In the distal hook region of both the lateral and median oral plates, the bone is especially dense and shows the highest bone volume fractions of the whole oral plate. The high bone density in the hook might be an adaption to the mechanical load applied in the distal part of the oral plate under a mechanical function scenario. Bone remodels (see Fig. 2H and 3H) and increases its density to withstand the load applied on the oral plate hook and the resulting stress patterns. Our results are compatible with bone remodelling under applied loads and indicate that the microstructure of the heterostracan lateral and median oral plates is adapted to a mechanical function.

In the lateral oral plate, the negative correlation between bone volume fraction and minimum principal stress is significant, but becomes non-significant when accounting for spatial autocorrelation, i.e. the fact that raster volumes located closer together show more similar values for bone volume fraction and minimum principal stress respectively. Spatial autocorrelation is generally expected for heterostracan oral plates. They are a naturally interconnected structure and thus we do not expect a random distribution of bone volume fraction and minimum principal stress values. In the median oral plate, an overall correlation between bone volume fraction and minimum principal stress values is absent and correlation test results are generally non-significant. The different results in the median and lateral oral plates are driven by the relative volume of the shaft versus the hook. The lateral oral plate has a bigger shaft, that shows a negative correlation between bone volume fraction and minimum principal stress values. The negative correlation of the shaft values drives the overall correlation for the lateral oral plate while the smaller hook shows no correlation. In comparison to the lateral oral plate, the median oral plate has a much smaller shaft portion due to its geometry. In addition, we have excluded a significant part of the shaft due to exceedingly high stress values at the proximal end of the oral plate from our analysis, which can be attributed to the fact that the model is fixed in this area. This leaves fewer data points for the shaft and more for the hook, which shows no correlation. With the hook points dominating, no significant correlation is detectable for the median oral plate overall. We conclude that the different correlation test results for the lateral and median oral plates are mostly driven by the difference in oral plate geometries and the relative volume of the shaft versus the hook. However, both the lateral and the median oral plates show exceedingly high bone volume fraction values and evidence for remodelling in the distal hook, where the load is applied in a mechanical scenario.

Generally, we observe a distinct distribution of wear on the oral plates. Wear is not randomly distributed, as would be expected of post-mortem abrasion, but is concentrated in specific regions of the oral plate pointing to a mechanical function scenario. The oral plates are consistently abraded at the distal tips and at the denticles on the lateral faces. Abrasion also occurs on the tubercles on the aboral surface but is more pronounced in the lateral oral plates than the median oral plates.

Our results contrast with Purnell (2002) who rejected a mechanical function based on an absence of wear on the plates except for abrasion on the aboral side, as we have described (see Fig. 2C and 2E; also Denison 1973, 1968). These abrasion patterns indicate that the aboral surface of the plates made repeated contact with an abrasive substrate. The small denticles on the lateral sides of the oral plate hook in the specimens examined by Purnell (2002) are relatively pristine and do not exhibit evidence of breakage. We would expect at least some wear on these denticles if they came in contact with hard food stuffs or surfaces in a mechanical function scenario. However, analysis of a broader range of oral plates (Grohganz et al., 2023) has revealed that material described by Purnell (2002) is unrepresentative. There is a much wider morphological variety from the discrete oakleaf-shaped denticles described by Purnell (2002) to more elongate ridge-like denticles, but all anteriorly-facing. These structures ranged from sharply pointed to abraded (see Fig. 2G and 3G). The abraded denticles occur on the lateral faces of the oral plate hook, which supports a mechanical scenario with the hook of the oral plate coming into contact with abrasive materials. This is also supported by the abrasion patterns we observe on the distal tips (see Fig. 2F and Fig. 3F, rectangles). They probably acted as the contact points of the oral plates with abrasive material in a mechanical scenario. Increased bone density in the hook domain and remodelling of the internal structure further indicate an adaption to mechanical loading. We observed evidence of remodelling processes in the internal microstructure of the oral plate hook (in both lateral and median plates) in the form of vascular spaces truncating existing growth layers within bone (see Fig. 2H and 3H).

The aboral surface is more worn in the lateral oral plates than in the median plates (see Fig. 2C and 2E, and 3C and 3E) because the lateral oral plates have a longer shaft and articulate directly with the postoral plate (Fig. 1A). This would have brought them into direct contact with the substrate when the mouth was open. The median oral plates sit in the middle of the apparatus and cannot have articulated directly with the postoral plate given their proportionally short shafts and the alignment of their distal tips with the longer, lateral plates (Fig. 1A). The oral plates must, therefore, have been held in soft tissue that spanned the width of the oral apparatus. This interpretation is supported by the observation that the oral surfaces of the plates bear open vascular canals (Fig. 2D). The median plate cannot easily have made contact with the sediment since there is a lack of wear on their aboral surface.

What mechanical feeding function is most likely for the heterostracan oral apparatus? Kiaer (1928) argued for predation, with the oral plates actively biting or crushing prey, analogous to gnathostome jaws. Several authors have proposed a scavenging feeding mode for heterostracans, with the oral plates similar in arrangement and function to the hagfish feeding apparatus (Janvier, 1974; Jarvik, 1980; Stensiö, 1932). In this scenario the oral plates would have fanned out to rasp or scrape against dead prey and then retracted until the distal hooks came into contact. The mouth of *Pteraspis* has also been interpreted as protrusible, used for selective detritus feeding with the oral plates acting as a sediment scoop (White, 1935).

All these scenarios (biting against the rostrum, rasping or scooping up detritus with the most distal part of the oral plates) would put a load on the hook and lead to the observed wear patterns on the distal tip, remodelling of the internal microstructure and increased bone volume fraction values in response to imposed load. We may reject the predation scenario, as the abrasion on the aboral surface is difficult to reconcile with a nektonic predatory lifestyle and indicates a rather specific mode of locomotion and feeding in which the aboral surface of the organism made contact with an abrasive substrate. This points towards a demersal lifestyle, moving over the seafloor to search for food. Feeding from material on the seafloor by scavenging or scooping best explains the observed abrasion on the aboral surface. However, the latero-ventral position of the eyes in heterostracans is in contrast with the dorsally placed eyes of contemporaneous osteostracans and galeaspids which are assumed to be deposit feeders (Gai et al., 2011; Janvier, 1985, 1996). In addition, the elongated snout commonly associated with pteraspids might have restricted the ability of the animal to place its mouth in contact with the substrate. But it might have also played a role in mechanical feeding, e.g. by stirring up sediment. Mechanical function hypotheses of the rostral process in pteraspids can be further tested with the method proposed for the oral plates in this paper. Nevertheless, the viability of the different mechanical function interpretations ultimately depends on the degree to which heterostracans could open their mouth. The scavenging scenario relies on a wider opening angle than the scooping scenario. Existing interpretations suggest that the degree to which heterostracans could open their mouth may have been limited (Halstead, 1973; Mallatt, 1984, 1996; Romer, 1959, 1966, 1970; Dearden et al., 2024). Recent advances in our knowledge of heterostracan oral anatomy provide us with a detailed reconstruction of the architecture and articulation of heterostracan tooth plates, including the original three-dimensional geometry of the oral plates and their relative arrangement in vivo (Dearden et al., 2024). These reconstructions allow us to further test competing mechanical function hypotheses, for example by constraining the oral plate movement with Range of motion (ROM) analyses.

Our results have broader implications for early vertebrate evolutionary scenarios like the New Head (Gans & Northcutt, 1983) and New Mouth (Mallatt, 1996) hypotheses. These hypotheses argue for increasingly active food acquisition and a development from passive suspension feeding towards active predation over time in early vertebrates. However, our results contradict these scenarios in showing that heterostracans, as one of the earliest stem-gnathostomes, probably already possessed an active mode of feeding, such as deposit feeding or scavenging. Our study highlights the importance of detailed knowledge of the feeding mode of early vertebrates, as it enables us to better test macroevolutionary scenarios like the New Head and New Mouth hypothesis and elucidate early vertebrate evolution.

## Conclusion

To test early vertebrate macroevolutionary scenarios, we need detailed knowledge of the feeding mode of early vertebrates. However, the feeding mode of early vertebrates including heterostracans still remains highly debated. We use Finite Element Analysis (FEA) and microstructure analysis to test a mechanical function scenario of the heterostracan oral plates. We show that minimum principal stress is correlated with bone volume fraction; the higher the absolute stress values, the higher the bone volume fraction values. This relationship shows most clearly in the lateral oral plate, whereas the median oral plate lacks a significant relationship due to its difference in oral plate geometry and its relatively smaller volume of the shaft in comparison to the hook. In the distal hook we find the overall highest bone volume fraction values and evidence for internal remodelling processes indicating an adaption of the microstructure to a mechanical loading at the distal hook. We propose that the heterostracan oral plates most likely performed a mechanical function.

The observed wear patterns on the aboral oral plate surface indicate a bottom-dwelling feeding mode for heterostracans, such as deposit feeding or scavenging; more active feeding modes than proposed by early vertebrate macroevolutionary scenarios for this basal group. Further testing requires detailed reconstructions of the oral plate apparatus, on which we can base Range of motion (ROM) analyses to determine the maximum possible opening angle. Applying modern computational techniques enables us to effectively test feeding mode hypotheses of early vertebrates for the first time. But further research is needed to elucidate the feeding mode of other early vertebrate groups and comprehensively test macroevolutionary scenarios like the New Head hypothesis or New Mouth hypothesis.

## Supplementary Material

Supplementary Materials
