# Supplementary material for: Finite element and microstructural analyses indicate that pteraspid heterostracan oral plate microstructure was adapted to a mechanical function
Source: Palaeontology. Author manuscript; Available in PMC 2025 Aug 21. (PMC7618031; doi:10.1111/pala.12733)
Supplement: Supplementary Materials [file EMS207764-supplement-Supplementary_Materials.pdf]

White, E. I. (1935). The ostracoderm Pteraspis Kner and the relationships of the agnathous vertebrates. *Philosophical Transactions of the Royal Society of London. Series B, Biological Sciences*, *225*(527), 381-457. https://doi.org/10.1098/rstb.1935.0015

Witten, P. E., & Huysseune, A. (2009). A comparative view on mechanisms and functions of skeletal remodelling in teleost fish, with special emphasis on osteoclasts and their function. *Biological Reviews*, *84*(2), 315-346. https://doi.org/10.1111/j.1469-185X.2009.00077.x

Wolff, J. (1892). *Das Gesetz der Transformation der Knochen. (Transl. The Law of Bone Remodelling)*. Springer-Verlag.

SUPPORTING INFORMATION

Data S1. Details on Synchrotron Radiation X-ray Tomography Microscopy (srXTM) measurements

srXTM investigations were performed at the X02DA TOMCAT beamline of the Swiss Light Source, Paul Scherrer Institute (Villigen, Switzerland) following a standard acquisition approach with the rotation axis located in the middle of the field of view and the acquisition of 1501 projections equiangularly distributed over 180° of rotation. Scan details are as follows: for specimens NHMUK PV P 43711, 4× objective with exposure time of 300 ms at 21 keV and resulting isotropic voxel dimensions of 1.625 um; for specimen NHMUK PV P 76697, 4x objective with exposure time of 120 ms at 21 keV and resulting isotropic voxel dimensions of 1.625 um. Projections were post-processed and rearranged into flat- and dark-field-corrected sinograms, and reconstruction was performed on a 60-core Linux PC farm. Slice data derived from the scans were analysed and manipulated using Avizo software for computed tomography, at the University of Bristol.

Data S2. Results of convergence tests for lateral oral plate model NHMUK PV P 43711

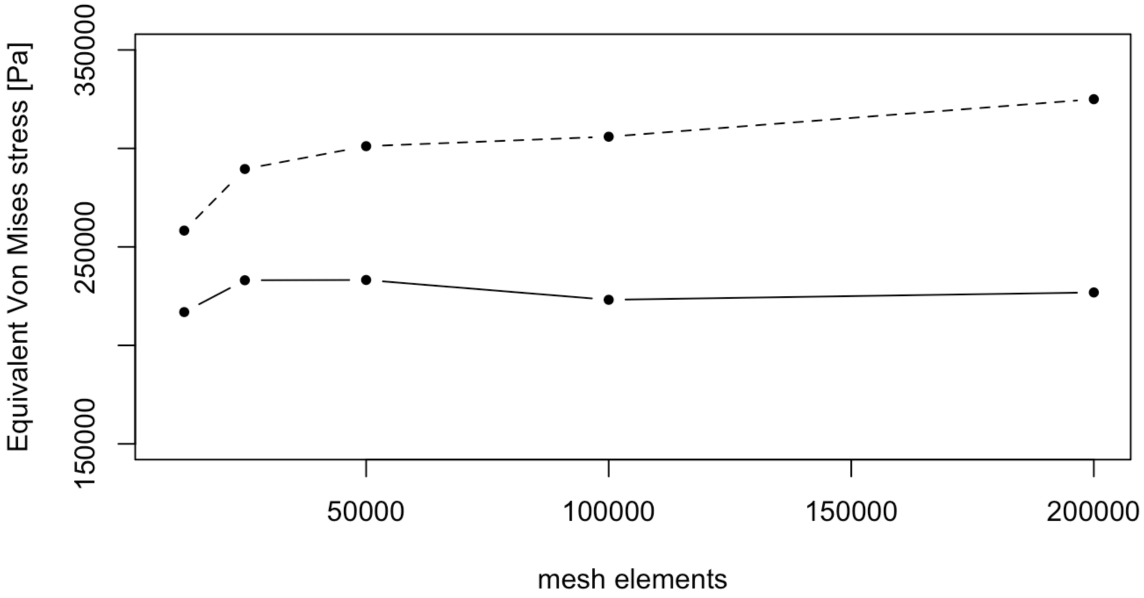

Number of mesh elements plotted against Equivalent Von Mises stress to check for an independence of the stress value results of changing mesh element numbers. Solid line represents mesh-weighted arithmetic mean (MWAM), dashed line represents arithmetic mean (AM).

Data S3. Results of the FE analyses of lateral oral plate sample NHMUK PV P 43711 with different loads and angles of attack

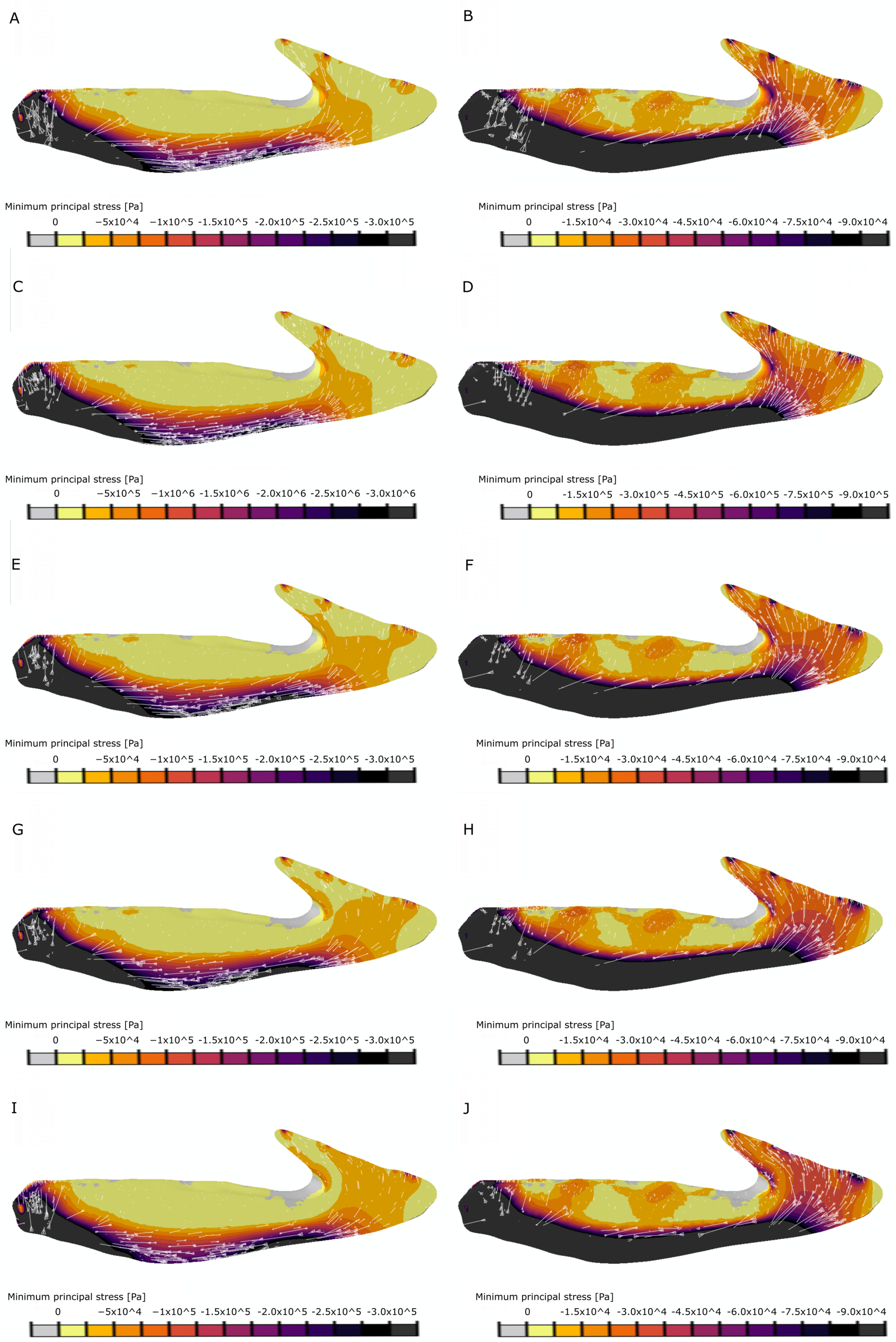

A-B, minimum principal stress patterns under applied load of 10N with different visualisation thresholds to optimally image the shaft and hook region; C-D, minimum principal stress patterns under applied load of 100N with different visualisation thresholds to optimally image the shaft and hook region; E-F, minimum principal stress patterns under angle of attack of 15 deg with different visualisation thresholds to optimally image the shaft and hook region; G-H, minimum principal stress patterns under angle of attack of 30 deg with different visualisation thresholds to optimally image the shaft and hook region; I-J, minimum principal stress patterns under angle of attack of 45 deg with different visualisation thresholds to optimally image the shaft and hook region.

Data S4. Results of the FE analyses and minimum principal stress – bone volume fraction correlation of the volume-scaled median oral plate sample NHMUK PV P 76697

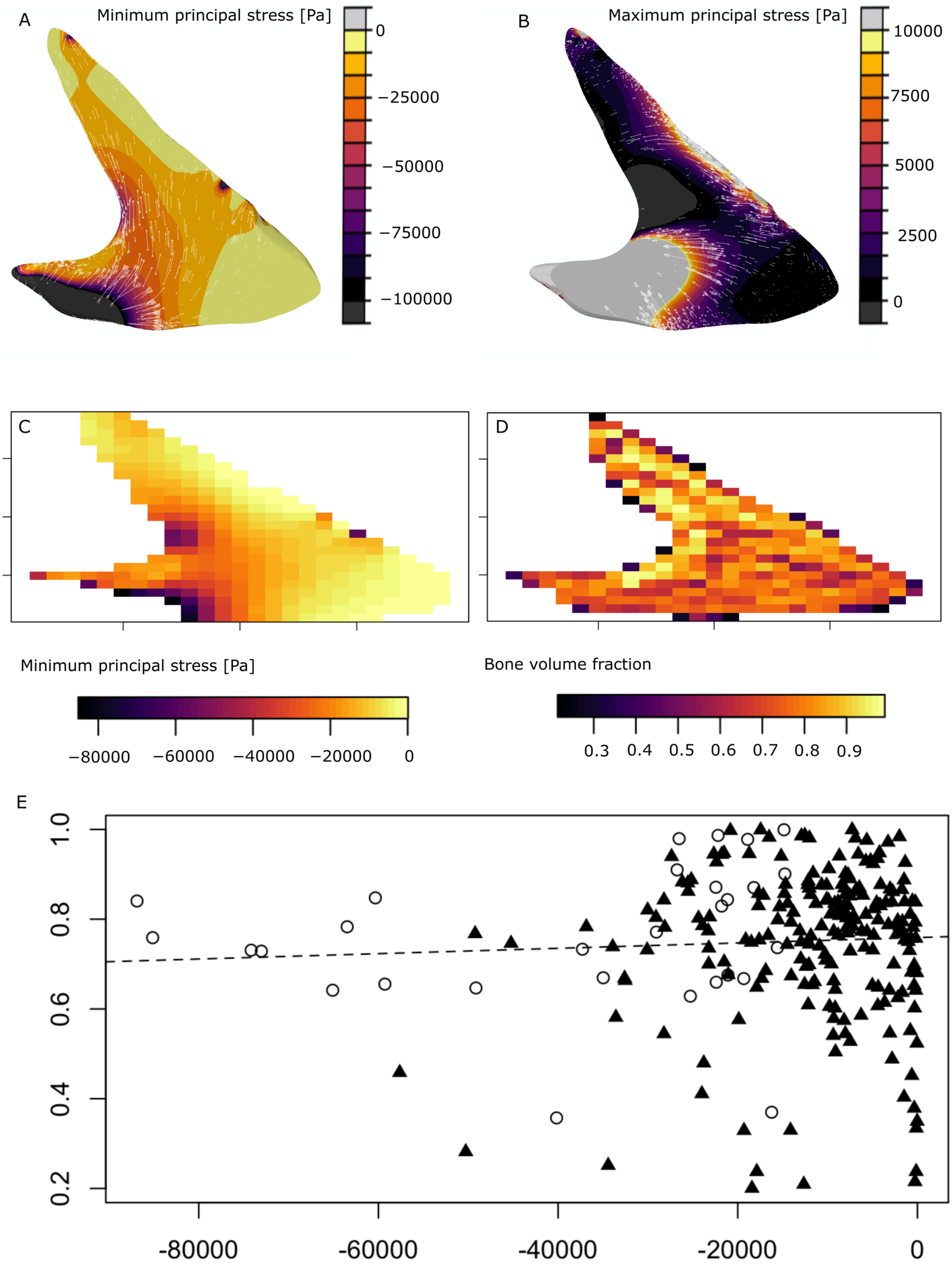

A, minimum principal stress patterns, with minimum principal stress increasing towards the bottom of the legend and stress trajectories as white arrows; B, maximum principal stress patterns, with maximum principal stress increasing towards the top of the legend and stress trajectories as white arrows; C, rastered FEA results for minimum principal stress; D, rastered results for bone volume fraction values; E, bone volume fraction raster means plotted against minimum principal stress raster means [Pa]; circles indicate raster volumes in the shaft of the oral plate, triangles in the hook of the oral plate; the dotted line represents the linear relationship between the two variables, bone volume fraction and minimum principal stress.
